# Immune Determinants of Viral Clearance in Hospitalised COVID-19 Patients: Reduced Circulating Naïve CD4+ T Cell Counts Correspond with Delayed Viral Clearance

**DOI:** 10.3390/cells11172743

**Published:** 2022-09-02

**Authors:** Mihaela Zlei, Igor A. Sidorov, Simone A. Joosten, Mirjam H. M. Heemskerk, Sebenzile K. Myeni, Cilia R. Pothast, Caroline S. de Brouwer, A. Linda Boomaars-van der Zanden, Krista E. van Meijgaarden, Shessy T. Morales, Els Wessels, Jacqueline J. Janse, Jelle J. Goeman, Christa M. Cobbaert, Aloys C. M. Kroes, Suzanne C. Cannegieter, Meta Roestenberg, Leonardus G. Visser, Marjolein Kikkert, Mariet C. W. Feltkamp, Sesmu M. Arbous, Frank J. T. Staal, Tom H. M. Ottenhoff, Jacques J. M. van Dongen, Anna H. E. Roukens, Jutte J. C. de Vries, in collaboration with BEAT-COVID, in collaboration with LUMC COVID

**Affiliations:** 1 Department of Immunology, Leiden University Medical Center, 2333 ZA Leiden, The Netherlands; 2 Clinical Microbiological Laboratory, Department of Medical Microbiology, Leiden University Medical Center, 2333 ZA Leiden, The Netherlands; 3 Department of Infectious Diseases, Leiden University Medical Center, 2333 ZA Leiden, The Netherlands; 4 Department of Hematology, Leiden University Medical Center, 2333 ZA Leiden, The Netherlands; 5 Molecular Virology Laboratory, Department of Medical Microbiology, Leiden University Medical Center, 2333 ZA Leiden, The Netherlands; 6 Department of Parasitology, Leiden University Medical Center, 2333 ZA Leiden, The Netherlands; 7 Medical Statistics Section, Department of Biomedical Data Sciences, Leiden University Medical Center, 2333 ZA Leiden, The Netherlands; 8 Department of Clinical Chemistry and Laboratory Medicine, Leiden University Medical Center, 2333 ZA Leiden, The Netherlands; 9 Department of Clinical Epidemiology, Leiden University Medical Center, 2333 ZA Leiden, The Netherlands; 10 Department of Intensive Care, Leiden University Medical Center, 2333 ZA Leiden, The Netherlands

**Keywords:** COVID-19, naïve CD4+ T cell, viral clearance

## Abstract

Virus-specific cellular and humoral responses are major determinants for protection from critical illness after SARS-CoV-2 infection. However, the magnitude of the contribution of each of the components to viral clearance remains unclear. Here, we studied the timing of viral clearance in relation to 122 immune parameters in 102 hospitalised patients with moderate and severe COVID-19 in a longitudinal design. Delayed viral clearance was associated with more severe disease and was associated with higher levels of SARS-CoV-2-specific (neutralising) antibodies over time, increased numbers of neutrophils, monocytes, basophils, and a range of pro-inflammatory cyto-/chemokines illustrating ongoing, partially Th2 dominating, immune activation. In contrast, early viral clearance and less critical illness correlated with the peak of neutralising antibodies, higher levels of CD4 T cells, and in particular naïve CD4+ T cells, suggesting their role in early control of SARS-CoV-2 possibly by proving appropriate B cell help. Higher counts of naïve CD4+ T cells also correlated with lower levels of MIF, IL-9, and TNF-beta, suggesting an indirect role in averting prolonged virus-induced tissue damage. Collectively, our data show that naïve CD4+ T cell play a critical role in rapid viral T cell control, obviating aberrant antibody and cytokine profiles and disease deterioration. These data may help in guiding risk stratification for severe COVID-19.

## 1. Introduction

SARS-CoV-2 viral load is considered an important determinant of disease severity and mortality [1,2,3]. Viral load peaks around symptom onset and declines afterwards, with a slower rate of decline in older patients [4,5]. Disease severity is affected by extensive pulmonary inflammation which plays a critical role in COVID-19 pathogenesis [6,7,8]. Though related, it remains to be established to what extent virus persistence drives ongoing tissue damage [9]. Identification of accurate correlates of protection against SARS-CoV-2 infection remains a critical challenge, and most studies focused on the magnitude of spike-specific antibody response or neutralising titer [10,11], supported by human challenge experiments with seasonal coronavirus infections [6,12]. Much less attention has been given to the magnitude or functional profile of cellular immune responses, in particular the naïve cellular subset [13].

The anti-SARS-CoV-2 immune response involves a highly organised cellular sequence of reactions in most individuals [13]. Shortly after infection the innate immune system sends out a rapid antiviral response through type I interferons, cytokines (such as IL-1, IL-18, and IL-6), and chemokines (such as CCL2 and CCL7) to inhibit virus replication [14]. Thereafter, adaptive immunity is activated. T lymphocytes play a crucial role in virus clearance after virus infection, whereas B lymphocytes mainly play a role by producing antibodies and neutralising viruses. T lymphocytes directly destroy infected cells to eliminate viruses and secrete cytokines to enhance T lymphocytes’ immune response and other immunocompetent cells, such as macrophages and B lymphocytes. Then, the body downregulates innate immunity to avoid nonspecific damage to the host. In some individuals, such a productive adaptive T and B cell response is not sufficiently mounted, leading to hyper inflammation mostly by innate immune cells, for instance local neutrophil invasion into the lung interstitium.

To date, it remains insufficiently clear to what extend the anticipated correlates of protection from infection after vaccination apply as correlates of SARS-CoV-2 clearance once infected. An extensive set of over 100 immune parameters collected longitudinally was studied in relation to timing to viral clearance. The timing of viral clearance was analysed in relation to the peaks of the humoral and cellular responses, and all immune parameters in this study were analysed in relation to each other. Based on these analyses, we propose a key role for naïve CD4 T cells for averting pathophysiological and immunological associations, in terms of mechanistic correlates of protection from severe clinical disease.

## 2. Materials and Methods

### 2.1. Experimental Design

To assess the relation of cellular, humoral, innate and adaptive immunological parameters with the timing of viral clearance, an integrated analysis was performed of viral and 122 immunological parameters in 102 hospitalised COVID-19 patients that were sampled longitudinally. Correlation was analysed for all immune parameters in relation to each other, and for the patient groups early versus delayed viral clearance.

### 2.2. Patients and Sample Collection

A total of 573 respiratory samples (thrice a week, nasopharyngeal swabs, and tracheal aspirates from intubated patients) and 333 blood samples (twice a week) were obtained from 102 COVID-19 patients with informed consent hospitalised in the Leiden University Medical Center (LUMC), from March 2020 to December 2020 (Wuhan-like viruses circulating, < 1% alpha variant until Jan 2021 nationally). Inclusion criteria were: admission at the LUMC, SARS-CoV-2 PCR positive, and minimum 18 years old. Exclusion criteria were: no informed consent from the patient or a representative. All participants were unvaccinated. From each patient the daily disease severity was scored. The severity score (range 0–17) includes the following parameters: respiratory rate, peripheral oxygen saturation on room air, P/F ratio^2^, oxygen flow, FiO_2_, Glasgow coma scale score, urea, and C reactive protein (Supplementary Table S6). The study was approved by the Medical Ethical Committee Leiden Delft Den Haag (NL73740.058.20) and registered in the Dutch Trial Registry (NL8589). Due to practical restrictions based on the extensive nature and/or biosafety aspects of the different immunoassays, subsets of patients were selected for the different types of analyses based on more narrow windows of patient enrollment. No selection was performed based on the duration of hospitalisation or time of symptom onset.

### 2.3. SARS-CoV-2 RT-PCR and Definition of Viral Clearance

In total 573 respiratory samples from 102 COVID-19 positive patients were tested by PCR. After extraction of nucleic acids from 200 μl sample using a MagNa Pure 96 instrument (Roche Diagnostics), ten microliters extract was used for SARS-CoV-2 E-gene detection by real-time reverse-transcription PCR on a CFX96 PCR instrument (Bio-Rad, Hercules, US): 50 °C for 5 min, followed by 95 °C for 20 s and then 45 cycles of 95 °C for 3 s, 55 °C for 10 s, and 60 °C for 30 s using primers and probe described by Corman et al. (2020 [15]). Viral clearance was defined as the last positive PCR available (Ct <= 40), either ≤ 21 or > 21 days post onset of symptoms prior to admission, the latter was considered delayed viral clearance [16]. Different thresholds for viral clearance were considered, 21 days was found to be more discriminative in the current dataset with a relative high proportion of ICU patients (see result section). The median difference between the last positive PCR results and first (consistent) negative PCR result was 3 days. Excluded from viral clearance analyses were patients with fatal outcome ≤ 21 days (*n* = 5), and patients that were transferred to another hospital ≤ 21 days (*n* = 6), resulting in 91 patients with viral clearance data available for statistical analyses.

### 2.4. Immune Parameter Analyses—SARS-CoV-2 Antibody Assays

In total 208 sera from 102 COVID-19 positive patients were tested for SARS-CoV-2-specific antibodies, sample numbers available per assay are included in the figure legends. Semi-quantitative detection of SARS-CoV-2 anti-nucleocapsid (N) protein IgG and anti-RBD of the S protein IgM antibodies was performed on the Abbott Architect platform [17,18]. In this antibody chemiluminescent microparticle immunoassay (CMIA) test, the SARS-CoV-2 antigen coated paramagnetic microparticles bind to the IgG, respectively, IgM antibodies that attach to the viral nucleocapsid protein in human serum samples. The Sample/Calibrator index values of chemiluminescence in relative light units (RLU) of 1.40 (IgG assay) respectively 1.00 (IgM assay) and above were considered as positive per the manufacturer’s instructions.

SARS-CoV-2 IgG antibody responses against the N-terminal (N-NT, position amino acids 1–246) and C-terminal (N-CT, position amino acids 181–419) antigen were analysed in a microparticle immunoassay. N full-length of human coronavirus 229E was included as a specificity control (N-229E). In brief, as previously described previously [19,20], viral gene fragments (gBlocks; IDT, San Jose, CA, USA) were cloned into pGEX-5x-3 vector (GE Healthcare Life Sciences, Chicago, IL, USA) expressed in E. coli and coupled to independent colour-coded magnetic bead sets (Bio-rad Laboratories, Hercules, CA, USA), to allow distinction between the antigens. Serum samples, 1:100 diluted, were pre-incubated with the antigen bead-mix for 1 h in blocking buffer and subsequently incubated with the antigen-coated bead sets for 1 h. For detection, biotinylated goat anti-human IgG (H+L) was used, followed by streptavidin-R-phycoerythrin. The phycoerythrin signal on each bead set was analysed in a Bioplex 200 analyser (Bio-Rad Laboratories, Hercules, CA, USA) and expressed as median fluorescence intensity (MFI).

Quantitative detection of SARS-CoV-2 anti-S1/S2 IgG antibodies was performed using the DiaSorin LIAISON platform. The CLIA assay consists of paramagnetic microparticles coated with distally biotinylated S1 and S2 fragments of the viral surface spike protein. RLUs proportional to the sample’s anti-S1/S2 IgG levels are converted to arbitrary units (AU)/millilitre based on a standardised master curve.

Semi-quantitative detection of SARS-CoV-2 anti-RBD IgM antibodies was performed using the Wantai IgM-ELISA (CE-IVD) kit (Sanbio, Tokyo, Japan) [21]. Briefly, the IgM u-chain capture method was used to detect IgM antibodies using a double-antigen sandwich immunoassay using mammalian cell-expressed recombinant antigens containing the RBD of the spike protein of SARS-CoV-2 as the immobilised and horseradish peroxidase-conjugated antigen. Sample/Cut-off index OD values of 1 and higher were considered positive per the manufacturer’s instructions.

Semi-quantitative detection of SARS-CoV-2 anti-S1 IgA antibodies was performed using the Euroimmun IgA 2-step ELISA [22]. Ratio values of 1.1 and higher were considered positive per the manufacturer’s instructions.

### 2.5. Virus Neutralisation Assay

A selection of 52 sera from 102 COVID-19 positive patients were tested for virus neutralising antibodies given the extensive nature and biosafety aspects of the assay. The patients were included in an early phase of the study. Neutralisation assays against live SARS-CoV-2 were performed using the microneutralisation assay previously described by Algaissi and Hashem [23]. Vero-E6 cells [CRL-1580, American Type Culture Collection (ATCC)] were grown in Eagle’s minimal essential medium (EMEM; Lonza) supplemented with 8% foetal calf serum (FCS; Bodinco BV, Alkmaar, the Netherlands), 1% penicillin-streptomycin (Sigma–Aldrich, St. Louis, MO, USA, P4458) and 2 mM L-glutamine (PAA). Cells were maintained at 37 °C in a humidified atmosphere containing 5% CO_2_. Clinical isolate SARS-CoV-2/human/NLD/Leiden-0008/2020 was isolated from a nasopharyngeal sample and its characterisation will be described elsewhere (manuscript in preparation). The next-generation sequencing derived sequence of this virus isolate is available under GenBank accession number MT705206.1 (https://www.ncbi.nlm.nih.gov/nuccore/1864563703, accessed on 1 January 2022) and shows one mutation in the Leiden-0008 virus spike protein compared to the Wuhan spike protein sequence resulting in Asp > Gly at position 614 (D614G) of the Spike protein. In addition, several non-silent (C12846U and C18928U) and silent mutations (C241U, C3037U, and C1448U) in other genes were found. Isolate Leiden-0008 was propagated and titrated in Vero-E6 cells using the tissue culture infective dose 50 (TCID_50_) endpoint dilution method and the TCID50 was calculated by the Spearman–Kärber algorithm as described [24]. All work with live SARS-CoV-2 was performed in a biosafety level 3 facility at Leiden University Medical Centre.

Vero-E6 cells were seeded at 12,000 cells/well in 96-well tissue culture plates 1 day prior to infection. Heat-inactivated (30 min at 56 °C) serum samples were analysed in duplicate. The panel of sera were two-fold serially diluted in duplicate, with an initial dilution of 1:10 and a final dilution of 1:1280 in 60 μL EMEM medium supplemented with penicillin, streptomycin, 2 mM L-glutamine and 2% FCS. Diluted sera were mixed with equal volumes of 120 TCID_50_/60 µL Leiden-0001 virus and incubated for 1 h at 37 °C. The virus-serum mixtures were then added onto Vero-E6 cell monolayers and incubated at 37 °C in a humidified atmosphere with 5% CO_2_. Cells either unexposed to the virus or mixed with 120 TCID_50_/60 µL SARS-CoV-2 were used as negative (uninfected) and positive (infected) controls, respectively. At 3 days post-infection, cells were fixed and inactivated with 40 µL 37% formaldehyde/PBS solution/well overnight at 4 °C. The fixative was removed from cells and the clusters were stained with 50 µL/well crystal violet solution, incubated for 10 min and rinsed with water. Dried plates were evaluated for viral cytopathic effect. Neutralisation titer was calculated by dividing the number of positive wells with complete inhibition of the virus-induced cytopathogenic effect, by the number of replicates, and adding 2.5 to stabilise the calculated ratio. The neutralising antibody titer was defined as the log_2_ reciprocal of this value. A SARS-CoV-2 back-titration was included with each assay run to confirm that the dose of the used inoculum was within the acceptable range of 30 to 300 TCID_50_.

### 2.6. Circulating Leukocyte Counts by Flow Cytometry

In total 133 blood samples from 102 COVID-19 positive patients were available for flow cytometry analyses. Absolute counts of the main circulating leukocyte subsets (Supplementary Figure S4) were obtained based on an adapted standard protocol for peripheral blood sample processing for flow cytometry measurement (for detailed protocol see www.EuroFlow.org (accessed on 1 January 2020) [25]) using an optimised combination of markers for surface staining (Primary Immunodeficiency Orientation Tube: PIDOT, Cytognos, Salamanca, Spain, https://www.cytognos.com/products/pidot-primary-immunodeficiency-orientation-tube (accessed on 1 January 2022) [26,27]).

For a better separation of the circulating plasmablasts, CD38 was added to the PIDOT combination (Supplementary Table S7 for details of antibody clones used). In summary, the procedure consisted in the bulk lysis of erythrocytes in fresh (same day) peripheral blood samples and staining of 2.5 × 10^6^ leukocytes with reconstituted PIDOT lyophilised antibody cocktail (containing CD8 FITC, IgD FITC, CD16 PE, CD56 PE, CD4PerCPCy5.5, CD19 PeCy7, TCRgd PE-Cy7, CD3 APC, CD56 APC-C750) and drop in antibody cocktail (containing per test: 2 µL CD27 BV421, 2,5 µL CD45RA BV510, 2 µL CD38 BV605, 0,6 µL pure CD38) (Supplementary Table S7) in a final 100 µL staining volume. The data (at least 1 million events) were acquired on a 3-laser Cytek^®^Aurora instrument (Cytek Biosciences, Fremont, CA, USA) at the Flow cytometry Core Facility (FCF) of Leiden University Medical Center (LUMC) in Leiden, Netherlands (https://www.lumc.nl/research/facilities/fcf, accessed on 1 January 2022). For data analysis, the Infinicyt software (Cytognos SL, Salamanca, Spain) was used. The cell subtypes identified and their expression profiles reflecting the gating strategy used are presented in Supplementary Figure S4. The absolute counts per µL fresh blood were determined by a double platform approach, using the absolute fresh leukocyte counts determined prior sample processing with hematological analyser (Sysmex) to the Statistics Configure tool of the Infinicyt software.

### 2.7. SARS-CoV-2-Specific T Cells

In total 132 blood samples from 102 COVID-19 positive patients were available for analyses of SARS-CoV-2-specific T cells. Blood was collected in CPT tubes (BD Biosciences, cat#362753) from which PBMCs were isolated using Ficoll-Isopaque. After cryopreservation, 1 × 10^6^ PBMCs were cultured in IMDM (Lonza, Basel, Switzerland, cat#BE12-722F) supplemented with 10% FCS (Sigma–Aldrich, cat#F7524), 1.4% L-glutamine (Lonza, cat#BE17-605E), and 1% Pen/Strep (Lonza, cat#DE17-602E). T cells were stimulated with 1 µg/mL SARS-CoV-2 peptide pool covering nucleocapsid (Miltenyi, Solothurn, Switzerland, cat#130-126-699), membrane (Miltenyi, cat#130-126-703), and immunodominant regions of the spike protein (Miltenyi, cat#130-126-701). Either 1% DMSO (Merck, Darmstadt, Germany, cat#1029311000) or 1 µg/mL CMV pp65 peptide pool were used as negative and positive control, respectively. After one hour incubation, 5 µg/mL Brefeldin A (Sigma–Aldrich) was added and after 16-hour incubation, the PBMCs were stained using Zombie-Red (Biolegend, San Diego, CA, USA, cat#423110), CD4-Pe-Cy7 (Beckman Coulter, Brea, CA, US, cat#737660), and CD8-APC-H7 (BD Biosciences, Haryana, India, cat#560179) for 30 min at 4 °C. This step was followed by an 8-minute 1% paraformaldehyde fixation at room temperature and subsequent ermeabilization with 0.1% saponin (Sigma–Aldrich) for 20 min at 4 °C. Intracellular staining was performed with an antibody mix containing the following antibodies: CD137-APC (BD Pharmingen, cat#550890), CD154-Pacific Blue (Biolegend, cat#310820), and IFNγ-BV711 (BD Biosciences, cat#564039) in 0.1% saponin for 30 min at 4 °C. After staining, the cells were resuspended in 0.1% saponin and measured on a 5-laser Cytek Aurora. The analysis was performed using FlowJo V10.7.1. (BD Biosiences, Haryana, India), in short: the lymphocytes were gated based on FSC-A and FSC-H. Zombie-Red negative cells were considered alive and T cell subsets were defined by expression of CD4 or CD8. Finally, activated CD4+ and CD8+ T cells were gated based on CD154+CD137+ of total CD4+ and CD137+IFNγ+ of total CD8+ T cells. SARS-CoV-2 CD4+ or CD8+ frequencies were calculated by subtracting the background (DMSO) and taking the sum of the three stimulations (= (activated in S% − activated in DMSO%) + (activated in M% − activated in DMSO%) + (activated in N% − activated in DMSO%)).

### 2.8. Cytokine/Chemokine Measurements

In total 333 sera from 102 COVID-19 positive patients were available for cytokine/chemokine measurements. Cytokines and chemokines were measured in serum by bead based multiplex assays using the BioPLex 100 system (Bio-Rad, Hercules, CA, USA) for acquisition as previously described [28]. Standard curves were provided with kits and a pooled sample of 4 COVID-19 patients was included as internal reference in all assays. Four commercially available kits were used Bio-Plex Pro™ Human Cytokine Screening Panel 48-plex; Bio-Plex Pro^tm^ Human Chemokine Panel 40-Plex; Bio-Plex Pro^tm^ Human Inflammation Panel 1, 37-Plex; Bio-Plex Pro^tm^ Human Th17 panel (IL-17F, IL-21, IL-23, IL-25, IL-31, IL-33) (all from Bio-Rad, Veenendaal, The Netherlands).

### 2.9. Statistical Analysis

Median with inter-quartiles range (IQR) were used to report continuous variables visualised in violine plots. Groups (early versus late clearance, non-ICU versus ICU) were compared with non-parametric Mann–Whitney U test. Correlations were analysed using Spearman’s rank correlation coefficient rho for potential non-linear correlations. Imputation for missing data (death or transfer within 21 days) was not performed, cases discharged to home within 21 days were considered early clearance and included in rank correlation analyses. Semiparametric regression of longitudinal data presented in this project was performed using R package mgcv, cubic spline were used with basis dimension value 8. Comparison of nonlinear regressions for groups of data was performed with gam.grptest R package (N.boot = 2000, m = 255) an interval of 100 days was used for comparing splines [29,30]. In spline figures, per-group trends were calculated using spline regression with bootstrap confidence intervals.

Tests with *p*-values ≤ 0.05 were considered statistically significant. Hierarchical clustering of correlation values were produced for all immune parameters (maximum level per patient) in relation to each other, and for the groups early versus delayed viral clearance, using Pearson’s correlation coefficient. Multiple testing correction of confidence values was performed using false discovery rate (FDR, Benjamini–Hochberg correction [31]). Statistical analyses were performed using statistical libraries of R (Spearman’s rank and Pearson correlation coefficients, heatmaps with hierarchical clustering, violine plots) and Perl (Mann–Whitney U test).

## 3. Results

### 3.1. Viral Clearance Preceded Critical Illness

**Figure 1:**
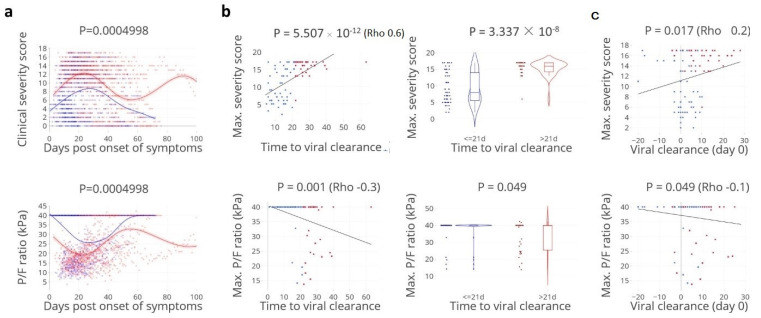
**Time to viral clearance correlates with disease severity.** (**a**). Kinetics of daily clinical severity scores and PO_2_/FiO_2_ (P/F) ratios: kinetics in relation to the time post onset of symptoms in days, per patient per groups with rapid: ≤ 21 days (blue lines/dots) and delayed: > 21 days viral clearance (red lines/dots). Lines indicate non-linear group trends. Spline regression with bootstrap confidence intervals, 39.9 kPa stands for the minimum disease severity. (**b**). The maximum (max.) daily clinical severity score and P/F ratio per patient in relation to time to viral clearance (days), defined as the last positive PCR. Spearman’s rank correlation (rho), and Mann–Whitney U test (violin plots). (**c**). **Viral clearance precedes critical illness.** Number of days between viral clearance (vertical line, day 0) in relation to the maximum severity scores and to P/F ratio, respectively. Viral clearance ≤ 21 days (blue lines/dots)/> 21 days (red lines/dot). Spearman’s rank correlation (rho), *n* = 91 patients.

To understand the relationship between disease severity, viral clearance, and concomitant immune responses, a prospective study was performed enrolling 102 hospitalised patients with moderate to severe COVID-19, of whom in total 91 had PCR data available to estimate viral clearance (see methods). Using a longitudinal follow-up, we analysed an extensive set of virological, immunological, and clinical parameters including a daily disease severity score (see method section). Both magnitude and timing of disease severity were studied in relation to the viral clearance from the respiratory tract, defined as the time from the onset of symptoms prior to admission to the last positive SARS-CoV-2 PCR result, available for 91 patients. Patients were median 67 years of age, 76% were male, the majority were ICU admitted (ratio ICU/non-ICU of 54 versus 37 patients, 60%), and the median time to viral clearance was 17 days (IQR 11–26 days). Demographics and underlying illness are listed in Supplementary Table S1. Time to viral clearance was significantly correlated with overall disease severity. Patients with delayed viral clearance, defined as viral clearance requiring > 21 days post onset of symptoms [16], had significantly higher maximum daily severity scores (Figure 1a,b). In the majority of the patients, episodes of most critical illness were observed after viral clearance occurred (Figure 1c). Since critical illness could be related to pulmonary and non-pulmonary factors, the PO_2_/FiO_2_ ratio was analysed here as well, with the same result: pulmonary illness persisted after SARS-CoV-2 had been cleared, possibly reflecting ongoing immune pathology at this stage of the disease. A comparable result was found when the ratio of arterial oxygen pressure to fractional inspired oxygen (PO_2_/FiO_2_) was used as a discriminating parameter for pulmonary illness in ventilated patients.

**Figure 2:**
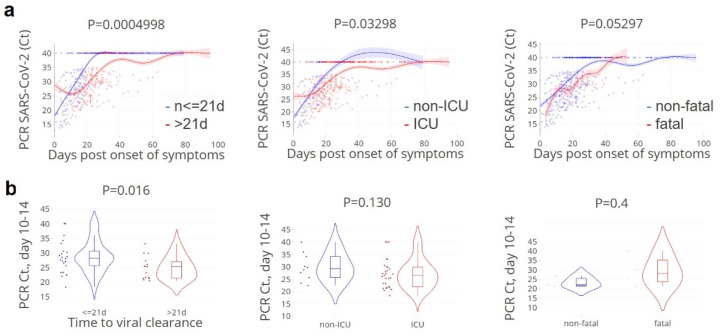
**Higher SARS-CoV-2 loads, over time are associated with ICU admission and fatality.** (**a**). kinetics in relation to the time post onset of symptoms (days), per patient per groups with rapid: ≤ 21 days (blue lines/dots) and delayed: > 21 days viral clearance (red lines/dots), ICU admission, and fatality. Lines indicate non-linear group trends. Spline regression with bootstrap confidence intervals. (**b**). Mean datapoints at day 10–14 data per patient, grouped according to the time to viral clearance ≤ 21 days (blue)/> 21 days (red), ICU admittance, and fatality, respectively. *n* = 91 patients. Mann–Whitney U test.

As anticipated, lower SARS-CoV-2 PCR Ct-values (higher viral loads) at day 10–14 after onset of symptoms was significantly associated with time to viral clearance, but also with ICU admission and fatality (Figure 2).

### 3.2. Apparent Paradoxical Higher Neutralising Antibody Titers in Cases with Delayed Clearance

**Figure 3:**
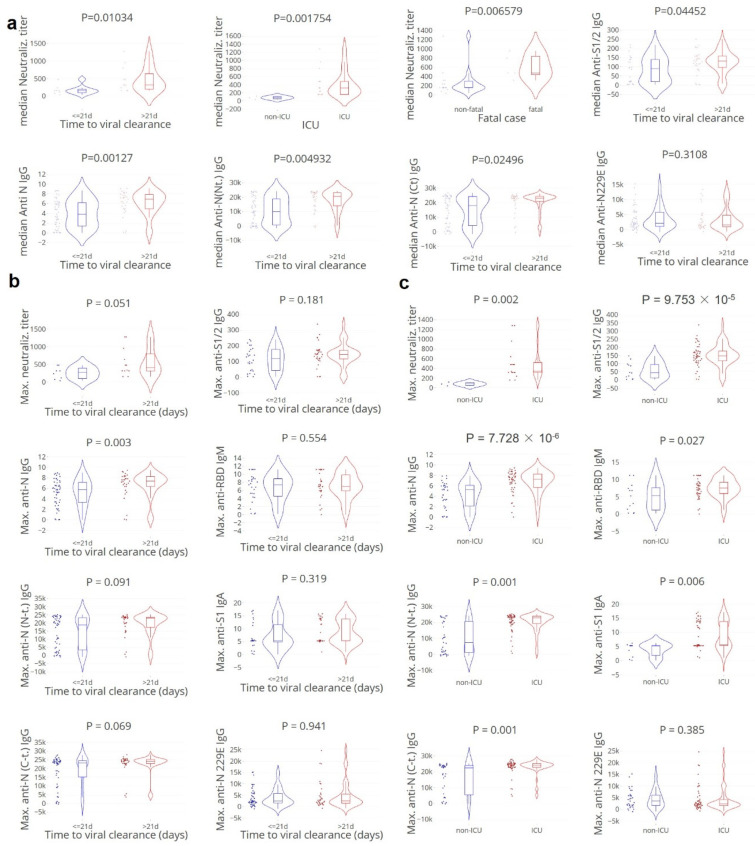
**Apparent paradoxical higher (neutralising) antibody titers in cases with delayed clearance, ICU admittance, and fatality. Anti-HCOV-229E IgG was included as negative control (see Supplementary Figure S1).** (**a**). Median values per patient grouped by the time to viral clearance: ≤ 21 (blue)/> 21 (red) days, ICU admittance, and fatality. Mann–Whitney U test. (**b**,**c**). Maximum values, grouped by the time to viral clearance: ≤21 (blue)/>21 (red) days, and ICU admittance. C-term./C-t.; C-terminal, N-term/N-t.; N-terminal. Neutralisation titer; 20 patients, 50 samples. Anti-S1/2 IgG; 58 patients, 158 samples. Anti-N IgG 82 patients, 196 samples. Anti-N(N/C terminus IgG and anti-N229 IgG; 84 patients, 187 samples. Mann–Whitney U test.

We first assessed to what extent anti-SARS-CoV-2-specific IgG and neutralising antibodies were correlated with viral clearance and protection from critical illness. Therefore, the kinetics and magnitude of SARS-CoV-2 IgG, IgA, and IgM responses to anti-spike (S), anti-receptor binding domain (RBD) of spike, and anti-nucleocapsid (N) proteins were analysed in relation to viral clearance (Figure 3 and Figure S1). Over time, the highest maximum levels of anti-N IgG, anti-S1/2 IgG, anti-RBD IgM, anti-S1 IgA, and also neutralising antibodies were observed in patients with delayed viral clearance (> 21 days) and more critical illness, which was confirmative of previous findings by others. No differences were found between the viral clearance groups for the negative control anti-HCoV-229E IgG levels.

### 3.3. Viral Clearance Dependent on (Naïve) T and B Cell Subsets

**Figure 4:**
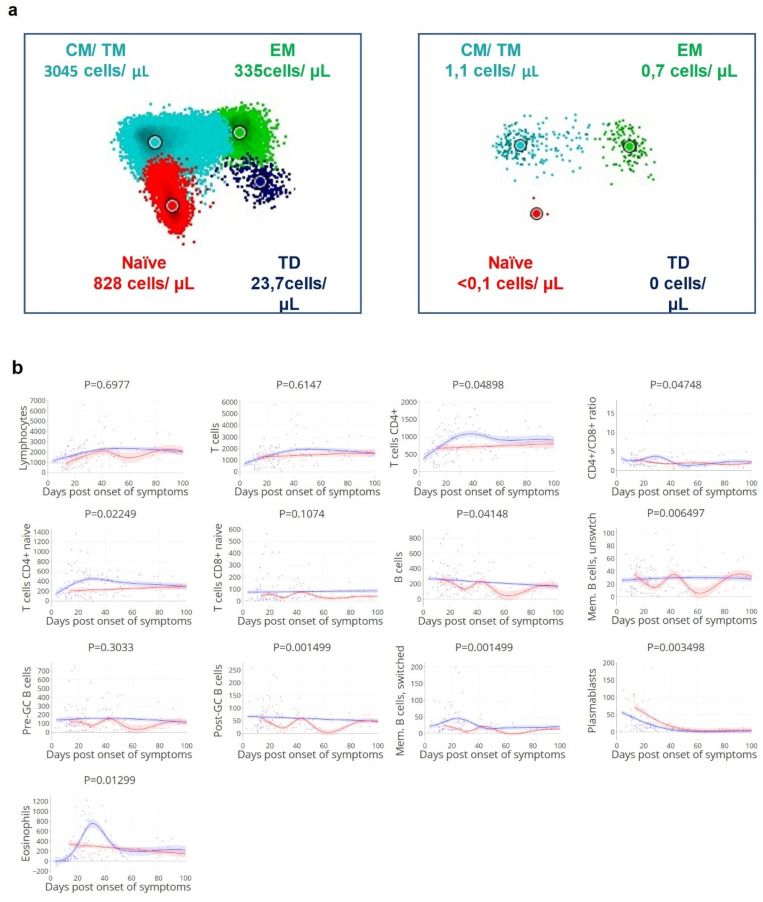

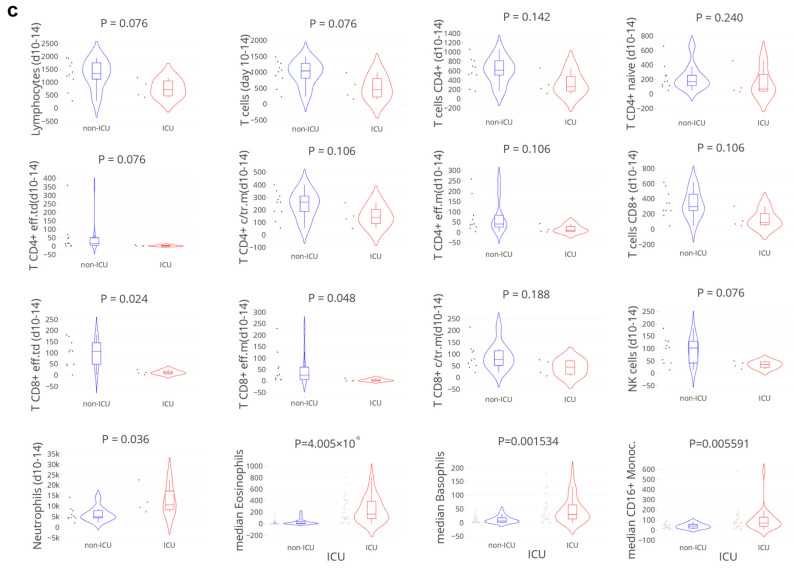
(**a**). **Low density of circulating CD4+ T cell compartment in a patient with delayed viral clearance (right).** Representative illustration of CD4+ T cell compartment in the peripheral blood samples of a patient with rapid (left: duration of positive PCR—1 day) or delayed viral clearance (right: duration of positive PCR—29 days). CM = central memory; TM = transitional memory; EM = effector memory; TD = effector, terminally differentiated; data analysis performed with Infinicyt software (Cytognos, Salamanca, Spain), each diagram is a representation of automated population separator (APS). One sample for each of the two patients. (**b**). **Reduced circulating (naïve) CD4+ T cells, post-GC B cells, and memory B cell count over time corresponds with delayed viral clearance**. Kinetics of absolute counts of circulating leukocyte subsets (measured as cells/µL) in relation to the time to viral clearance, a selection of subsets based on *p*-values (see Supplementary Figure S2). Groups with rapid: ≤ 21 days (blue lines/dots) and delayed: > 21 days viral clearance (red lines/dots). Lines indicate non-linear group trends. Spline regression with bootstrap confidence intervals. (**c**). **Reduced CD8+ effector T cell count at day 10–14 corresponds with ICU admission**. Mean values at day 10–14 grouped by non-ICU (blue)/ICU (red) patients, and median values per patient. T CD4+ c/tr.m; T cells CD4+ central/transitional memory, T CD4+ eff.m; T cells CD4+ effector memory, td; terminally differentiated, pre-GC; pre germinal center B cells; circulating naïve B cells [32]. *n* = 41 patients, 128 samples. Mann–Whitney U test.

Cellular immune responses to SARS-CoV-2 have been subject of study [13] but the magnitude of deviation of both indirect and direct effects of the different cellular components resulting in delayed SARS-CoV-2 clearance are still incompletely understood. To study the correlation between different T and B cell subsets with viral clearance and disease severity, subsets of immune cells over the disease course were analysed in correlation with the magnitude and timing of viral clearance (Figure 4 and Figure S2). We observed that delayed viral clearance was associated with reduced CD4+ T cell counts (Figure 4a,b), including CD4+ T cells, naïve CD4+ T cells (*p* 0.022), B cells, unswitched memory B cells, post-GC B cells, switched memory B cells, and plasmablasts. Patients with lower numbers of CD8+ T effector cells at day 10–14 post onset of symptoms had a higher risk of being admitted to ICU (Figure 4). These findings illustrate that both naïve and effector T and B cells are involved in early control of SARS-CoV-2.

### 3.4. Delayed Viral Clearance Corresponds with Significant Innate Cell Expansion

In contrast to the adaptive cellular responses, eosinophil counts were significantly increased in patients with delayed viral clearance (Figure 4b). Additionally, increased neutrophil, eosinophil, basophil, and CD16+ monocyte counts at day 10–14 were associated with ICU admission (Figure 4c). Despite these high innate cellular responses, SARS-CoV-2 infection could not be controlled in these patients at an early stage.

### 3.5. SARS-CoV-2-Specific CD4+ T Cell Fraction of Importance for Rapid Clearance

**Figure 5:**
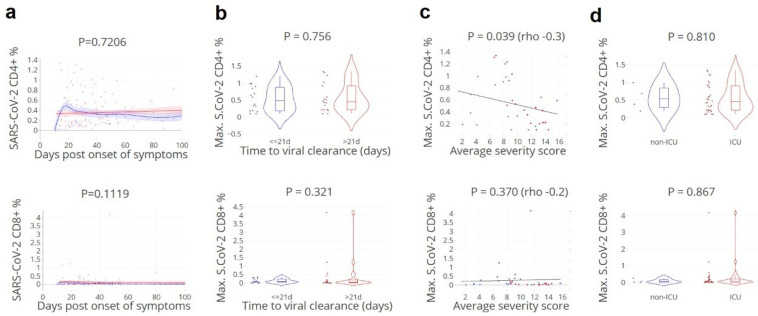
**Lower SARS-CoV-2-specific CD4+ T cell counts correspond with higher disease severity**. (**a**). Kinetics of circulating CD4+ and CD8+ T-cells specific to SARS-CoV-2 spike, nucleocapsid, and membrane peptides, in relation to the day of viral clearance. (**b**). Maximum values grouped by the time to viral clearance: ≤ 21 (blue)/> 21 (red) days. Lines indicate non-linear group trends. Spline regression with bootstrap confidence intervals. (**c**). Maximum values and average daily severity scores per patient, grouped by the time to viral clearance: ≤ 21 (blue)/> 21 (red) days. (**d**). Maximum values grouped by non-ICU (blue)/ICU (red) patients. SARS-CoV-2 CD4+ (%) = percentage of CD154+CD137+ out of total CD4+ T cells, SARS-CoV-2 CD8+ (%) = percentage of CD137+IFNg+ out of total CD8+ T cells. S.CoV-2; SARS-CoV-2. *n* = 34 patients, 130 samples. Mann–Whitney U test.

When analysing the proportion of circulating SARS-CoV-2-specific CD4+ and CD8+ T cells, a significant inverse association was found with the average daily severity score: a higher proportion of SARS-CoV-2-specific CD4+ T cells was found in patients with rapid viral clearance and lower severity score (Figure 5). This effect was not detected for specific IFNγ+ CD8+ T cells though the number of samples available for this specific assay was low.

### 3.6. Cytokine and Chemokine ‘Storms’ in Patients with Delayed Viral Clearance

**Figure 6:**
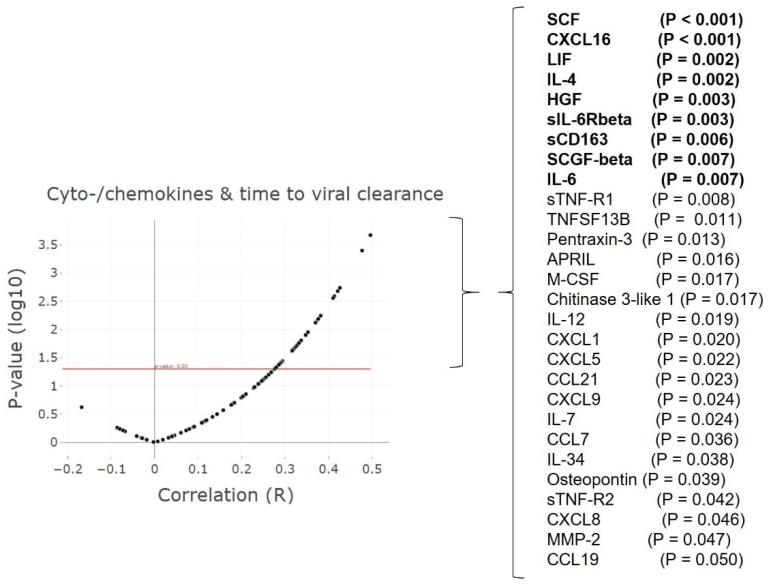
**Higher levels of pro-inflammatory cyto/chemokines correspond with delayed viral clearance.** Volcano plot: cytokines and chemokines (maximum levels) correlated with the time to viral clearance, a selection based on *p*-value ≤ 0.05, with R ≥ 0.4 in bold. An overview of all cytokines and chemokines assessed is listed in Supplementary Table S2. *n* = 51 patients, 321 samples. Spearman’s rank correlation (rho).

A large set of 83 cytokines and chemokines (Supplementary Table S2) was analysed in relation to the time to viral clearance (Figure 6). Twenty-eight cyto-/chemokines correlated significantly and positively with delayed viral clearance, markers with the strongest correlations (R > 0.4) included: IL-4, IL-6, sIL-6Rbeta, LIF, HGF, SCGF-beta, and sCD163. The strongest correlation was observed with SCF and CXCL16 (Supplementary Table S3). These cyto-/chemokines have mainly pro-inflammatory effects, indicating active inflammation. Macrophages and neutrophils are known to be among their main producing cells, corresponding with the increased number of neutrophil cells detected. Many of these cytokines and chemokines have been associated with chronic inflammatory conditions and extensive tissue remodelling including those related to the lung, but also with diseases associated with vascular abnormalities.

### 3.7. Timing of Rapid Clearance Coincides with T and B Cell Subsets Peaks

**Figure 7:**
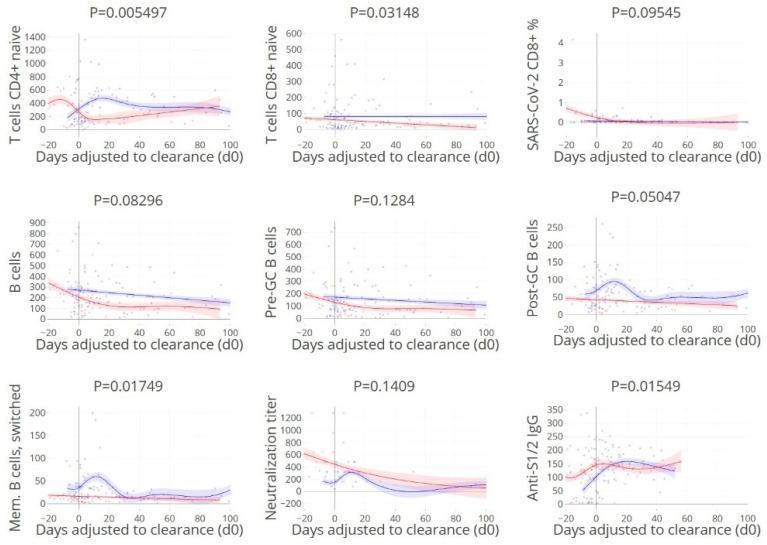
Peak levels of naïve CD4+ T cells, naïve CD8+ T cells, post GC and memory B cells, and anti-spike IgG coincide with rapid viral clearance. Kinetics of humoral and cellular immune parameters adjusted to the timing of viral clearance (day 0). A selection of humoral and cellular parameters was made based on findings described above. Blue: viral clearance ≤ 21 days, red: > 21 days. Lines indicate non-linear group trends. Spline regression with bootstrap confidence intervals.

Subsequently, the timing of viral clearance was studied in relation to the kinetics of humoral and cellular immune responses by adjustment of the time scale to the time of viral clearance (set at day 0) (Figure 7). We reasoned that if the peak of protective immune parameters would coincide with the timing of viral clearance, the association with viral clearance would be direct, as opposed to indirect. The timing of the peak levels of naïve CD4+ T cells, naïve CD8+ T cells, post GC and memory B cells, and anti-spike IgG coincided with the time of clearance, indicating a more direct role in virus control. Thus, timing of the peak (Figure 7), but not the absolute level of neutralising antibody levels (Figure 3) coincided with viral clearance (see above). These findings are supported by previous data suggesting that despite a critical role for virus neutralisation, viral spread by cell-to-cell contact may be resistant to antibody neutralisation [13,33].

### 3.8. Overall Matrix of Immune Parameters in Relation with Viral Clearance

**Figure 8:**
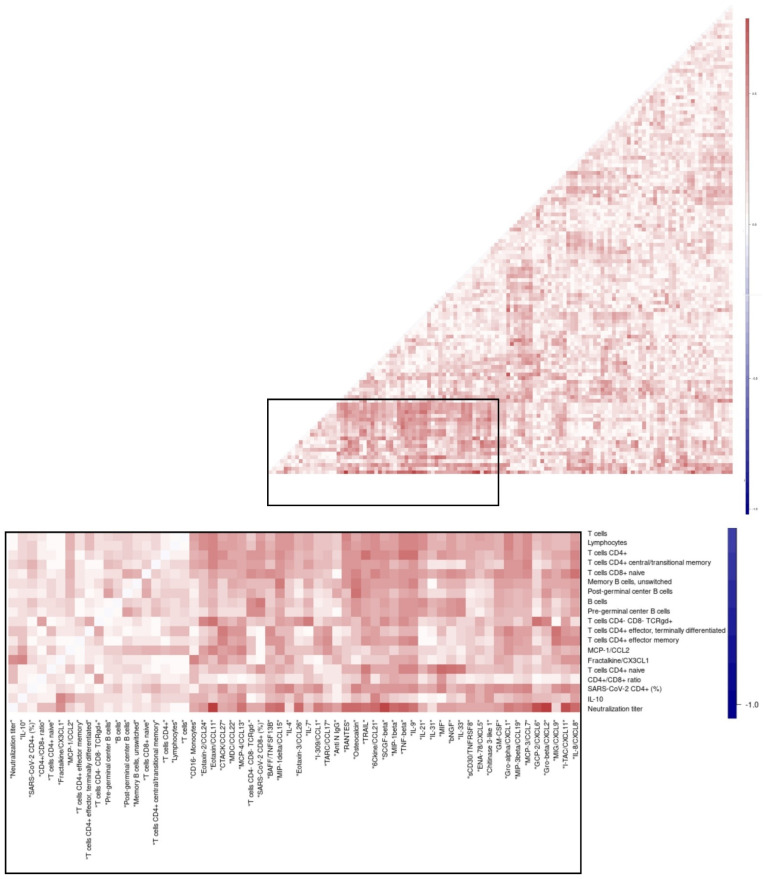
**CD4+ T cell subset counts inversely correlate with proinflammatory cytokines:** naïve CD4 T cells with MIF, and CD4+ T cells with IL-9/TNFbeta, in patients with rapid viral clearance. Correlation heatmaps of the maximum level of immune parameters in individual patients, for the differences between the patient groups with viral clearance > 21 days and clearance ≤ 21 days. MIF; macrophage migration inhibitory factor. Pearson’s correlation coefficient.

We hypothesised that immune parameters could be associated with each other due to related underlying biology, and, more specifically, that decreased numbers of CD4+ T cell subset counts would be inversely correlated with higher innate cell responses and cyto-chemokine levels in the patients with delayed clearance. This was assessed in a maximal broad exploration that was hypothesis-free and thus open for detection of any types of interactions by correlating the maximum levels of all immune parameters measured for all patients in a matrix (Figure 8, Supplementary Table S4). CD4+ T cell subset counts were associated with each other, such as naïve CD4+ T cell counts with total CD4+ T cell counts. When focussing on comparisons between immune parameters from different sub-compartments (cellular, humoral, soluble), significant positive correlations were observed between the proportion of SARS-CoV-2-specific CD8+ T cells and IL7/TNFSF13B. Additionally, matrices were designed separately for patients with delayed and rapid viral clearance (Supplementary Figure S3) and subsequently, the differences in R-values between these two groups were visualised (Figure 8, Supplementary Table S5). Differences detected between the clearance groups with significant *p*-values included a negative correlation for CD4+ T cells with IL-9/TNF-beta in the group with delayed clearance in contrast to a positive correlation with clearance ≤ 21 days. Similarly, in patients with delayed viral clearance, lower numbers of naïve CD4+ T cells were associated with higher concentrations of inflammatory cytokine macrophage inhibitory factor (MIF), a critical upstream mediator of innate immunity.

## 4. Discussion

Here, we describe the clearance of SARS-CoV-2 infection by using integrated analysis of longitudinal datasets including viral load data and > 100 immune parameters representing a plethora of adaptive and innate components of the immune system. This approach enabled analysis of the kinetics of immune parameters in parallel, in relation to the timing of viral clearance. Similarity matrices of the studied immune parameters in relation to each other allowed maximal broad and hypothesis-free identification of parameters that differed between cases with delayed viral clearance in comparison with clearance within 21 days.

Our data suggest that inefficient virus control is linked to impaired levels of several B and T cell subsets, with important associations for CD4+ effector cells and naïve CD4+ T cells. Previous studies have focused on T cell responses in hospitalised COVID-19 patients [34,35,36,37,38] but studies on naïve CD4 and CD8 T cells are scarce [39,40,41]. Neo-antigen-specific responses depend on the pool of naïve lymphocytes, and a small starting pool of naïve T cells may limit the likelihood of priming a fast or robust virus-specific T cell response due to the reduced starting repertoire in a limited naïve pool [39]. In future studies, the size of the naïve TCR repertoire which correlates with the pool size of CD4 T cells would be an interesting parameter to measure in COVID-19 clinical studies. Consistently, elderly individuals, who have a smaller naïve T cell pool, have decreased immune repertoires [42], making it harder to quickly mount an effective adaptive immune response to a neo-antigen such as SARS-CoV-2. Future studies may address specific details of the overall picture drawn in this paper, by zooming in on more specific interactions at higher resolution using more advanced statistical models and measures of TCR repertoire.

Delayed viral clearance was associated with increased disease severity which is in line with other reports [3], even though the highest level of critical illness usually occurred after patients had cleared SARS-CoV-2, illustrative of (lung) tissue damage-related immunopathology as the underlying mechanism in this phase of critical illness as opposed to ongoing viral replication-related tissue damage. This underscores the importance of rapid viral control, given the apparent association of delayed viral clearance with later disease severity. Since analysis of SARS-CoV-2 loads in patients in general is largely influenced by the time points analysed [3], we selected the time to viral clearance from onset of symptoms for the analyses of levels of immune parameters potentially involved.

**Figure 9:**
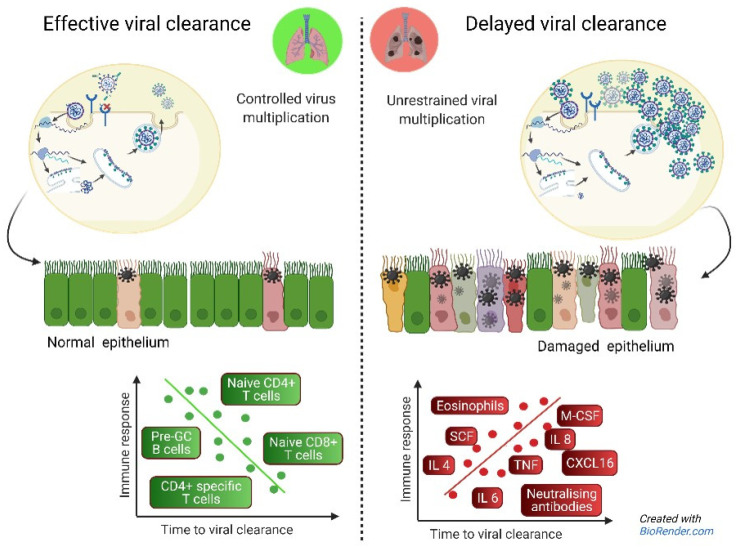
**Hypothesis-generated drawing** including all immune parameters in this study found to be associated with (delayed) viral clearance. Pre-GC; pre germinal center B cells; circulating naïve B cells [32]. Adapted from “Coronavirus Replication Cycle”, by BioRender.com, accessed 1 January 2022.

Apparent paradoxically, delayed viral clearance and disease severity were associated with higher maximum levels of SARS-CoV-2 neutralising antibodies over time. These findings are in line with other studies including patients and asymptomatic SARS-CoV-2 positive individuals, with the latter group having the lowest or even negative antibody titers, being at the other end of the spectrum of the disease [34,43,44,45,46]. This phenomenon ‘cellular sensitisation without seroconversion’ indicates a potential role for the cellular T cell-mediated immune system in clearing infection before it is fully established [13]. In contrast, patients with delayed viral clearance have prolonged exposure to viral antigens which can result in prolonged antibody responses until the cellular component is effective to clear the virus [34]. In addition, viral loads in prolonged infections are higher (as shown in this study) which in itself may trigger more intense B cellular responses. Moreover, once produced, antibodies may accumulate simply due to their half-life. It is of importance to realise that the analyses of the magnitude and timing of the peak of responses, and seropositivity early after onset [47] will give an incomplete picture when assessed as single variables, as can be seen in our neutralising antibody data: the peak coincides with clearance but the highest peaks are seen in cases with delayed clearance. While correlates of protection prior to viral infection in general are typically defined as cut-off levels for effective immune parameters, our findings suggest that these levels may not hold as predictors of viral clearance in infected patients. This may be explained by viral persistence as a driving force for the ongoing production and accumulation of antibodies, after the initial failure of rapid T and B cell control, early after infection (Figure 9).

These data were complemented by data on concomitant immunological responses and we observed a significant increase in cells and soluble mediators involved in innate immunity: neutrophils, monocytes, basophils, and the pro-inflammatory cyto-/chemokines Il-4, -6, sIL-6Rbeta, LIF, HGF, and SCGF-beta were all increased in patients with delayed viral clearance and, in most cases, with more severe illness. Some of these soluble components have been suggested by others as predictors of disease severity and fatal outcome [48,49,50], in line with our data. Our findings support the postulation that severe COVID-19 is preceded by an inefficient virus control by adaptive immunity, causing prolonged virus-induced (lung) tissue damage, which necessitates enhanced responses of the innate immune system to control and resolve the tissue damage. Clearly, such extensive innate responses are not sufficiently effective in all cases with severe COVID-19 disease courses. The antibody data in the current study are limited to measurement of systemic responses. Though nasal antibodies have been strongly correlated with serum antibodies in COVID-19 patients, this correlation was weaker for the mucosal antibody type IgA. Previous work showed that an early and higher nasal antibody response, in particular of the IgM and IgA type, was associated with lower viral loads [51]. Tissue localisation of specific isotypes of antibodies may explain a lack of association with clearance when measuring systemic responses only.

A caveat on the interpretation of these data is the fact that we only had access to peripheral blood to analyse the cellular response. It is possible that the mucosal immune responses in the upper respiratory tract and lungs were more robust in patients with early viral clearance. It is possible that the mucosal immune responses in the upper respiratory tract and lungs were more robust in patients with early viral clearance. An indication that this mucosal response in the nose is different from peripheral responses that can be readily measured was recently reported [52] as there was no T lymphopenia in the nasal scrapes compared to peripheral blood. Yet these local responses are generated from naïve T cells, therefore we interpret the finding of lower naïve T cell numbers in more severely ill patients reported here, coupled with the lower number of SARS-specific CD4+ T cells, as biologically meaningful. This lower number of naïve T cells indicates a smaller naïve repertoire (none of these patients had been vaccinated because vaccines were not yet available during the time patients were recruited) and therefore a lower change of having a perfectly fitting TCR. It would be very interesting to investigate if formal TCR repertoire analyses would yield a more complete picture of a presumed smaller repertoire pool in more severely ill patients.

Another caveat of the study is the unavailability of data on viral clearance of patients that were lost to follow up within 21 days: fatal and discharged cases, which were excluded from analysis. Though the number of fatal cases excluded was relatively small (5 out of 102 patients), this may have resulted in bias towards less critical illness in the delayed clearance group and less discriminative power.

Higher maximum neutralisation titers correlated with higher levels of pro-inflammatory cytokines and chemokines in our integrated analysis. In patients with delayed viral clearance, levels of activated CD4+ T cells and IL-9 were inversely correlated as opposed to the positive correlation in patients with earlier clearance. Future studies are needed to address a direct or indirect interaction between these two and the role of an IL-9 secreting T cell subset known as Th9 cells.

In some reports, the increased proportion of SARS-CoV-2-specific CD4+ and CD8+ T cells seemed to correlate with disease severity whereas other reports do not see this correlation [12,46,53]. Although this pro-inflammatory profile may be an aggravating factor promoting immunopathogenesis, CD4+ T cells have also been demonstrated to control SARS, as depletion of these cells in mice resulted in delayed clearance of the virus, and more severe lung inflammation [7,54]. Here, we demonstrate that the proportion of SARS-CoV-2-specific CD4+ T cells is higher in patients that better control the virus, and that the peak of both SARS-CoV-2-specific CD4+ and CD8+ T cells correlated with the time of viral clearance, indicating a more direct role in virus control. As argued elsewhere [55], multiple high affinity antibodies are likely needed to block SARS-CoV-2 binding to ACE2 in the respiratory tract. For such antibody responses to occur, CD4+ T cell help is required to induce somatic hypermutation for increased affinity as well as class switch recombination to IgA antibodies, capable of crossing epithelial barriers. In some studies, late-responders were evaluated at a later stage after exposure, thus allowing more time to induce T cell memory [56]; indeed, in our study the highest numbers of SARS-CoV-2-specific CD8+ cells tended to be detected > 60 days after onset of symptoms, though this was not statistically significant. It may be that cytotoxic responses against SARS-CoV-2 infected epithelial cells, although clearing viral infection, also lead to local lung epithelial damage contributing to disease symptoms.

In conclusion, our data suggest that delayed viral clearance is associated with failure of rapid T and B cell control, possibly due to a more narrow repertoire of naïve CD4+ T cells, followed by prolonged viral exposure causing enhanced (lung) tissue damage, requiring enhanced responses of neutralising antibodies, chemokines, cytokines, and innate immune cells. Logically, progressive immunosenescence during aging explains why elderly are more vulnerable to infections with new microorganisms, that require control by an adaptive immune system with a highly diverse Ig and TCR repertoire. Our data suggest that correlates of protection from infection after vaccination [11] might be quantitatively distinct from correlates of clearance once infected, and can guide early risk stratification of patients and treatment strategies.

## Data Availability

All patient data are shared in a data warehouse (Opal), managed by the section Advanced Data Management and hosted on a secure server within the LUMC.

## Supplementary Materials

The following supporting information can be downloaded at: https://www.mdpi.com/article/10.3390/cells11172743/s1, Figure S1. Kinetics of SARS-CoV-2-specific (neutralising) antibody responses in hospitalised patients in relation to the time post onset of symptoms, per patient, with anti-HCOV-229E IgG as control. Blue: viral clearance ≤ 21 days, red > 21 days. C-term./C-t.; C-terminal, N-term/N-t.; N-terminal. Figure S2. Kinetics of absolute counts of circulating leukocyte subsets (measured as cells/µL) in relation to the time post onset of symptoms, per patient. Blue: viral clearance ≤ 21 days, red: > 21 days. Figure S3. Correlation heatmaps of the max. level of immune parameters in individual patients, per group: a, viral clearance ≤ 21 days and b, viral clearance > 21 days. Figure S4. Leukocyte subtypes identified by flow cytometry and their expression profiles reflecting the gating strategy used. A: APS (Automatic Population Separator) plots that use the information from all the parameters included in the file; B: Bi-parameter plots reflecting the expression profile for each of the markers used (Far-left: major leukocyte subsets, middle-left: major lymphocyte subsets, middle right: main T cell subsets; far-right: main B cell sunsets; DCs = dendritic cells, EM = effector memory cells, Eff = terminally differentiated effector cells, NK = natural killer, TM = transitional memory cells. Table S1. Demographic and underlying illness from the 91 hospitalised SARS-CoV-2 patients with viral clearance data available. Table S2. List of immune parameters measured in this study. Table S3. List of cytokines significantly (*p* < 0.05) related to time to viral clearance after adjustment for multiple testing. Table S4. List of significant correlations (R > 0.7) between the maximum level of all immune parameters with each other (all patients). Table S5. List of differences between the correlations of the max. level of the immune parameters of the group’s viral clearance ≤21 days and >21 days. A selection is made for differences between R values of the clearance groups of >0.5, and with significant *p*-values for both variables. Table S6. Daily severity score parameters. Table S7. List of reagents used for the identification of B and T cell subsets; NA—not applicable.

## References

[1] Fajnzylber J., Regan J., Coxen K., Corry H., Wong C., Rosenthal A., Worrall D., Giguel F., Piechocka-Trocha A., Atyeo C. (2020). SARS-CoV-2 viral load is associated with increased disease severity and mortality. Nat. Commun..

[2] Westblade L.F., Brar G., Pinheiro L.C., Paidoussis D., Rajan M., Martin P., Goyal P., Sepulveda J.L., Zhang L., George G. (2020). SARS-CoV-2 Viral Load Predicts Mortality in Patients with and without Cancer Who Are Hospitalized with COVID-19. Cancer Cell.

[3] Zheng S., Fan J., Yu F., Feng B., Lou B., Zou Q., Xie G., Lin S., Wang R., Yang X. (2020). Viral load dynamics and disease severity in patients infected with SARS-CoV-2 in Zhejiang province, China, January-March 2020: Retrospective cohort study. BMJ.

[4] He X., Lau E.H.Y., Wu P., Deng X., Wang J., Hao X., Lau Y.C., Wong J.Y., Guan Y., Tan X. (2020). Temporal dynamics in viral shedding and transmissibility of COVID-19. Nat. Med..

[5] Neant N., Lingas G., Le Hingrat Q., Ghosn J., Engelmann I., Lepiller Q., Gaymard A., Ferre V., Hartard C., Plantier J.C. (2021). Modeling SARS-CoV-2 viral kinetics and association with mortality in hospitalized patients from the French COVID cohort. Proc. Natl. Acad. Sci. USA.

[6] Huang A.T., Garcia-Carreras B., Hitchings M.D.T., Yang B., Katzelnick L.C., Rattigan S.M., Borgert B.A., Moreno C.A., Solomon B.D., Trimmer-Smith L. (2020). A systematic review of antibody mediated immunity to coronaviruses: Kinetics, correlates of protection, and association with severity. Nat. Commun..

[7] Tay M.Z., Poh C.M., Renia L., MacAry P.A., Ng L.F.P. (2020). The trinity of COVID-19: Immunity, inflammation and intervention. Nat. Rev. Immunol..

[8] Yang L., Liu S., Liu J., Zhang Z., Wan X., Huang B., Chen Y., Zhang Y. (2020). COVID-19: Immunopathogenesis and Immunotherapeutics. Signal Transduct. Target. Ther..

[9] Schurink B., Roos E., Radonic T., Barbe E., Bouman C.S.C., de Boer H.H., de Bree G.J., Bulle E.B., Aronica E.M., Florquin S. (2020). Viral presence and immunopathology in patients with lethal COVID-19: A prospective autopsy cohort study. Lancet Microbe.

[10] Earle K.A., Ambrosino D.M., Fiore-Gartland A., Goldblatt D., Gilbert P.B., Siber G.R., Dull P., Plotkin S.A. (2021). Evidence for antibody as a protective correlate for COVID-19 vaccines. Vaccine.

[11] Khoury D.S., Cromer D., Reynaldi A., Schlub T.E., Wheatley A.K., Juno J.A., Subbarao K., Kent S.J., Triccas J.A., Davenport M.P. (2021). Neutralizing antibody levels are highly predictive of immune protection from symptomatic SARS-CoV-2 infection. Nat. Med..

[12] Le Bert N., Tan A.T., Kunasegaran K., Tham C.Y.L., Hafezi M., Chia A., Chng M.H.Y., Lin M., Tan N., Linster M. (2020). SARS-CoV-2-specific T cell immunity in cases of COVID-19 and SARS, and uninfected controls. Nature.

[13] Moss P. (2022). The T cell immune response against SARS-CoV-2. Nat. Immunol..

[14] Diamond M.S., Kanneganti T.D. (2022). Innate immunity: The first line of defense against SARS-CoV-2. Nat. Immunol..

[15] Corman V.M., Landt O., Kaiser M., Molenkamp R., Meijer A., Chu D.K., Bleicker T., Brünink S., Schneider J., Schmidt M.L. (2020). Detection of 2019 novel coronavirus (2019-nCoV) by real-time RT-PCR. Eurosurveillance.

[16] Xue J., Zheng J., Shang X., Qin E., Zhao P., He Y., Liu M., Zhang J., Liu H., Bai C. (2020). Risk factors for prolonged viral clearance in adult patients with COVID-19 in Beijing, China: A prospective observational study. Int. Immunopharmacol..

[17] Escribano P., Alvarez-Uria A., Alonso R., Catalan P., Alcala L., Munoz P., Guinea J. (2020). Detection of SARS-CoV-2 antibodies is insufficient for the diagnosis of active or cured COVID-19. Sci. Rep..

[18] Maine G.N., Lao K.M., Krishnan S.M., Afolayan-Oloye O., Fatemi S., Kumar S., VanHorn L., Hurand A., Sykes E., Sun Q. (2020). Longitudinal characterization of the IgM and IgG humoral response in symptomatic COVID-19 patients using the Abbott Architect. J. Clin. Virol..

[19] Kamminga S., van der Meijden E., Wunderink H.F., Touze A., Zaaijer H.L., Feltkamp M.C.W. (2018). Development and Evaluation of a Broad Bead-Based Multiplex Immunoassay to Measure IgG Seroreactivity against Human Polyomaviruses. J. Clin. Microbiol..

[20] van der Meijden E., Kazem S., Burgers M.M., Janssens R., Bouwes Bavinck J.N., de Melker H., Feltkamp M.C. (2011). Seroprevalence of trichodysplasia spinulosa-associated polyomavirus. Emerg. Infect. Dis..

[21] Zhao J., Yuan Q., Wang H., Liu W., Liao X., Su Y., Wang X., Yuan J., Li T., Li J. (2020). Antibody Responses to SARS-CoV-2 in Patients With Novel Coronavirus Disease 2019. Clin. Infect. Dis..

[22] Beavis K.G., Matushek S.M., Abeleda A.P.F., Bethel C., Hunt C., Gillen S., Moran A., Tesic V. (2020). Evaluation of the EUROIMMUN Anti-SARS-CoV-2 ELISA Assay for detection of IgA and IgG antibodies. J. Clin. Virol..

[23] Algaissi A., Hashem A.M. (2020). Evaluation of MERS-CoV Neutralizing Antibodies in Sera Using Live Virus Microneutralization Assay. Methods Mol. Biol..

[24] Killington J.C.H.a.R.A. (1996). Virus Isolation and Quantitation. Virology Methods Manual.

[25] van der Velden V.H.J., Flores-Montero J., Perez-Andres M., Martin-Ayuso M., Crespo O., Blanco E., Kalina T., Philippe J., Bonroy C., de Bie M. (2019). Optimization and testing of dried antibody tube: The EuroFlow LST and PIDOT tubes as examples. J. Immunol. Methods.

[26] Van Dongen J.J., Van der Burg M., Kalina T., Perez-Andres M., Mejstrikova E., Vlkova M., Lopez-Granados E., Wentink M., Kienzler A.K., Philippé J. (2019). EuroFlow-Based Flowcytometric Diagnostic Screening and Classification of Primary Immunodeficiencies of the Lymphoid System. Front. Immunol..

[27] Van der Burg M., Kalina T., Perez-Andres M., Vlkova M., Lopez-Granados E., Blanco E., Bonroy C., Sousa A.E., Kienzler A.K., Wentink M. (2019). The EuroFlow PID Orientation Tube for Flow Cytometric Diagnostic Screening of Primary Immunodeficiencies of the Lymphoid System. Front. Immunol..

[28] van Meijgaarden K.E., Khatri B., Smith S.G., Drittij A., de Paus R.A., Goeman J.J., Ho M.M., Dockrell H.M., McShane H., Joosten S.A. (2018). Cross-laboratory evaluation of multiplex bead assays including independent common reference standards for immunological monitoring of observational and interventional human studies. PLoS ONE.

[29] Zhao S., Bakoyannis G., Lourens S., Tu W.Z. (2020). Comparison of nonlinear curves and surfaces. Comput. Stat. Data Anal..

[30] Dette H., Schorning K. (2016). Optimal Designs for Comparing Curves. Ann. Stat..

[31] Benjamini Y.H.Y. (1995). Controlling the false discovery rate: A practical and powerful approach to multiple testing. J. R. Stat. Soc..

[32] Del Pino-Molina L., Lopez-Granados E., Lecrevisse Q., Torres Canizales J., Perez-Andres M., Blanco E., Wentink M., Bonroy C., Nechvatalova J., Milota T. (2020). Dissection of the Pre-Germinal Center B-Cell Maturation Pathway in Common Variable Immunodeficiency Based on Standardized Flow Cytometric EuroFlow Tools. Front. Immunol..

[33] Zeng C., Evans J.P., King T., Zheng Y.M., Oltz E.M., Whelan S.P.J., Saif L.J., Peeples M.E., Liu S.L. (2022). SARS-CoV-2 spreads through cell-to-cell transmission. Proc. Natl. Acad. Sci. USA.

[34] Tan A.T., Linster M., Tan C.W., Le Bert N., Chia W.N., Kunasegaran K., Zhuang Y., Tham C.Y.L., Chia A., Smith G.J.D. (2021). Early induction of functional SARS-CoV-2-specific T cells associates with rapid viral clearance and mild disease in COVID-19 patients. Cell Rep..

[35] Kedzierska K., Thomas P.G. (2022). Count on us: T cells in SARS-CoV-2 infection and vaccination. Cell Rep. Med..

[36] Mele D., Calastri A., Maiorano E., Cerino A., Sachs M., Oliviero B., Mantovani S., Baldanti F., Bruno R., Benazzo M. (2021). High Frequencies of Functional Virus-Specific CD4(+) T Cells in SARS-CoV-2 Subjects with Olfactory and Taste Disorders. Front. Immunol..

[37] Zhou R., To K.K., Wong Y.C., Liu L., Zhou B., Li X., Huang H., Mo Y., Luk T.Y., Lau T.T. (2020). Acute SARS-CoV-2 Infection Impairs Dendritic Cell and T Cell Responses. Immunity.

[38] Weiskopf D., Schmitz K.S., Raadsen M.P., Grifoni A., Okba N.M.A., Endeman H., van den Akker J.P.C., Molenkamp R., Koopmans M.P.G., van Gorp E.C.M. (2020). Phenotype and kinetics of SARS-CoV-2-specific T cells in COVID-19 patients with acute respiratory distress syndrome. Sci. Immunol..

[39] Rydyznski Moderbacher C., Ramirez S.I., Dan J.M., Grifoni A., Hastie K.M., Weiskopf D., Belanger S., Abbott R.K., Kim C., Choi J. (2020). Antigen-Specific Adaptive Immunity to SARS-CoV-2 in Acute COVID-19 and Associations with Age and Disease Severity. Cell.

[40] Gallerani E., Proietto D., Dallan B., Campagnaro M., Pacifico S., Albanese V., Marzola E., Marconi P., Caputo A., Appay V. (2021). Impaired Priming of SARS-CoV-2-Specific Naive CD8(+) T Cells in Older Subjects. Front. Immunol..

[41] Nguyen T.H.O., Rowntree L.C., Petersen J., Chua B.Y., Hensen L., Kedzierski L., van de Sandt C.E., Chaurasia P., Tan H.X., Habel J.R. (2021). CD8(+) T cells specific for an immunodominant SARS-CoV-2 nucleocapsid epitope display high naive precursor frequency and TCR promiscuity. Immunity.

[42] Britanova O.V., Putintseva E.V., Shugay M., Merzlyak E.M., Turchaninova M.A., Staroverov D.B., Bolotin D.A., Lukyanov S., Bogdanova E.A., Mamedov I.Z. (2014). Age-related decrease in TCR repertoire diversity measured with deep and normalized sequence profiling. J. Immunol..

[43] Seow J., Graham C., Merrick B., Acors S., Pickering S., Steel K.J.A., Hemmings O., O’Byrne A., Kouphou N., Galao R.P. (2020). Longitudinal observation and decline of neutralizing antibody responses in the three months following SARS-CoV-2 infection in humans. Nat. Microbiol..

[44] Legros V., Denolly S., Vogrig M., Boson B., Siret E., Rigaill J., Pillet S., Grattard F., Gonzalo S., Verhoeven P. (2021). A longitudinal study of SARS-CoV-2-infected patients reveals a high correlation between neutralizing antibodies and COVID-19 severity. Cell. Mol. Immunol..

[45] Jeewandara C., Jayathilaka D., Gomes L., Wijewickrama A., Narangoda E., Idampitiya D., Guruge D., Wijayamuni R., Manilgama S., Ogg G.S. (2021). SARS-CoV-2 neutralizing antibodies in patients with varying severity of acute COVID-19 illness. Sci. Rep..

[46] Poland G.A., Ovsyannikova I.G., Kennedy R.B. (2020). SARS-CoV-2 immunity: Review and applications to phase 3 vaccine candidates. Lancet.

[47] Dispinseri S., Secchi M., Pirillo M.F., Tolazzi M., Borghi M., Brigatti C., De Angelis M.L., Baratella M., Bazzigaluppi E., Venturi G. (2021). Neutralizing antibody responses to SARS-CoV-2 in symptomatic COVID-19 is persistent and critical for survival. Nat. Commun..

[48] Del Valle D.M., Kim-Schulze S., Huang H.H., Beckmann N.D., Nirenberg S., Wang B., Lavin Y., Swartz T.H., Madduri D., Stock A. (2020). An inflammatory cytokine signature predicts COVID-19 severity and survival. Nat. Med..

[49] Angioni R., Sanchez-Rodriguez R., Munari F., Bertoldi N., Arcidiacono D., Cavinato S., Marturano D., Zaramella A., Realdon S., Cattelan A. (2020). Age-severity matched cytokine profiling reveals specific signatures in COVID-19 patients. Cell Death Dis..

[50] Varchetta S., Mele D., Oliviero B., Mantovani S., Ludovisi S., Cerino A., Bruno R., Castelli A., Mosconi M., Vecchia M. (2021). Unique immunological profile in patients with COVID-19. Cell. Mol. Immunol..

[51] Fröberg J., Gillard J., Philipsen R., Lanke K., Rust J., van Tuijl D., Teelen K., Bousema T., Simonetti E., van der Gaast-de Jongh C. (2021). SARS-CoV-2 mucosal antibody development and persistence and their relation to viral load and COVID-19 symptoms. Nat. Commun..

[52] Roukens A.H., Pothast C.R., König M., Huisman W., Dalebout T., Tak T., Azimi S., Kruize Y., Hagedoorn R.S., Zlei M. (2022). Prolonged activation of nasal immune cell populations and development of tissue-resident SARS-CoV-2-specific CD8+ T cell responses following COVID-19. Nat. Immunol..

[53] Grifoni A., Weiskopf D., Ramirez S.I., Mateus J., Dan J.M., Moderbacher C.R., Rawlings S.A., Sutherland A., Premkumar L., Jadi R.S. (2020). Targets of T Cell Responses to SARS-CoV-2 Coronavirus in Humans with COVID-19 Disease and Unexposed Individuals. Cell.

[54] Chen J., Lau Y.F., Lamirande E.W., Paddock C.D., Bartlett J.H., Zaki S.R., Subbarao K. (2010). Cellular immune responses to severe acute respiratory syndrome coronavirus (SARS-CoV) infection in senescent BALB/c mice: CD4+ T cells are important in control of SARS-CoV infection. J. Virol..

[55] Khatri I., Staal F.J.T., van Dongen J.J.M. (2020). Blocking of the High-Affinity Interaction-Synapse Between SARS-CoV-2 Spike and Human ACE2 Proteins Likely Requires Multiple High-Affinity Antibodies: An Immune Perspective. Front. Immunol..

[56] Thieme C.J., Anft M., Paniskaki K., Blazquez-Navarro A., Doevelaar A., Seibert F.S., Hoelzer B., Konik M.J., Berger M.M., Brenner T. (2020). Robust T Cell Response Toward Spike, Membrane, and Nucleocapsid SARS-CoV-2 Proteins Is Not Associated with Recovery in Critical COVID-19 Patients. Cell. Rep. Med..

