# Supplementary material for: Immune Determinants of Viral Clearance in Hospitalised COVID-19 Patients: Reduced Circulating Naïve CD4+ T Cell Counts Correspond with Delayed Viral Clearance
Source: Cells. 2022 Sep 2;11(17):2743. doi: 10.3390/cells11172743 (PMC9455062; doi:10.3390/cells11172743)

Supplementary Figure S1. Kinetics of SARS-CoV-2 specific (neutralizing) antibody responses in hospitalized patients in relation to the time post onset of symptoms, per patient, with anti-HCOV-229E IgG as control. Blue: viral clearance ≤ 21 days, red > 21 days. C-term./C-t.; C-terminal, N-term/N-t.; N-terminal.

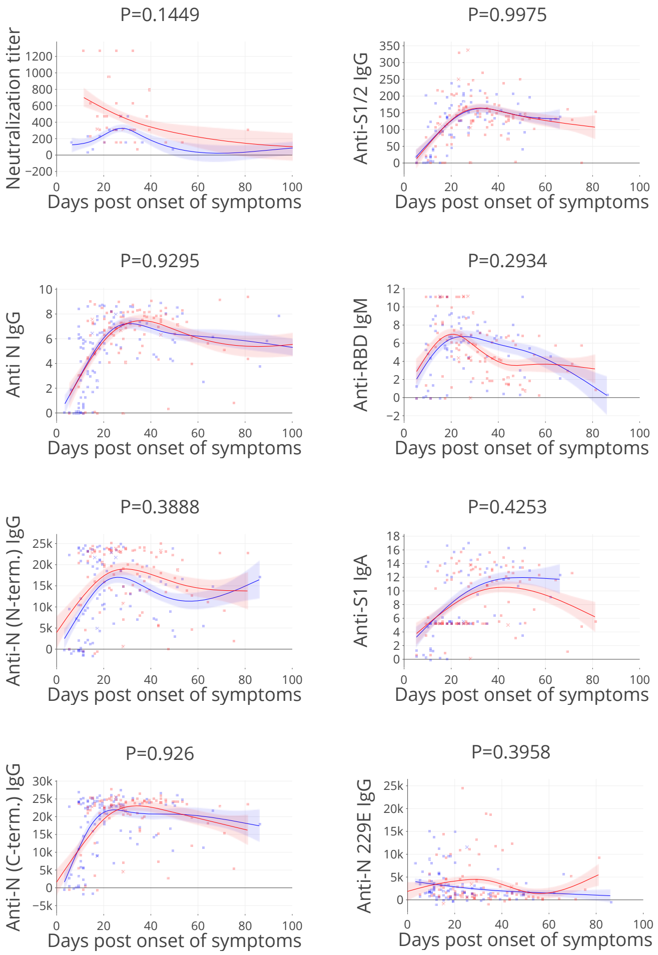

Supplementary Figure S2. Kinetics of absolute counts of circulating leukocyte subsets (measured as cells/ µL) in relation to the time post onset of symptoms, per patient. Blue: viral clearance ≤ 21 days, red: > 21 days. Analyses of max. values did not result in significant differences between the two groups of viral clearance, ICU and fatality.

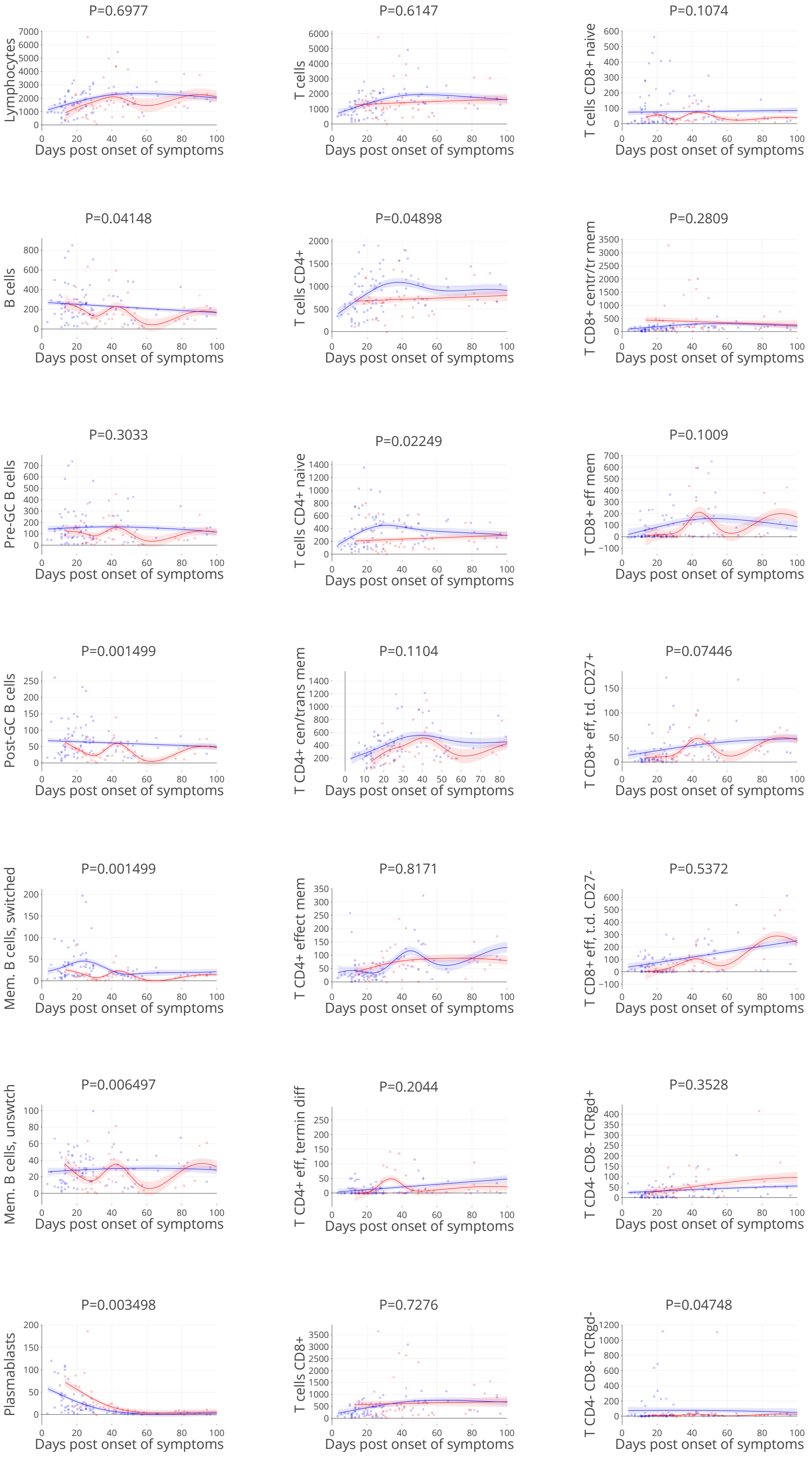

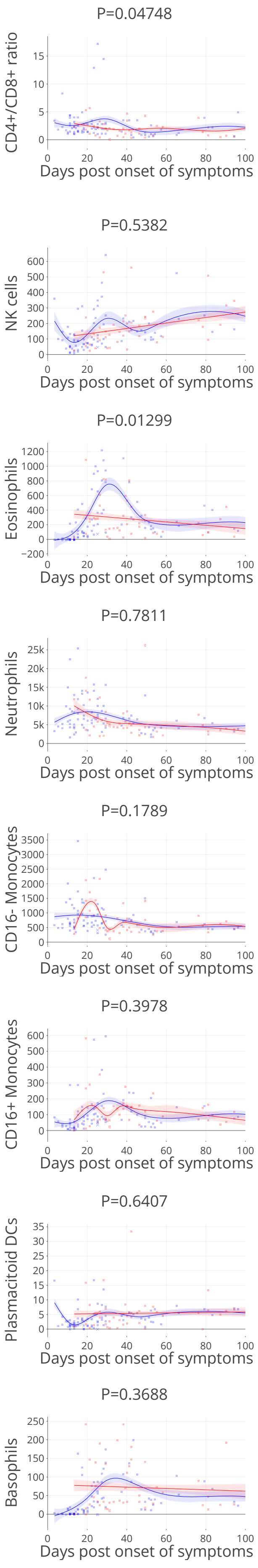
P=0.04748
CD4+/CD8+ ratio
Days post onset of symptoms
P=0.5382
NK cells
Days post onset of symptoms
P=0.01299
Eosinophils
Days post onset of symptoms
P=0.7811
Neutrophils
Days post onset of symptoms
P=0.1789
CD16- Monocytes
Days post onset of symptoms
P=0.3978
CD16+ Monocytes
Days post onset of symptoms
P=0.6407
Plasmacitoid DCs
Days post onset of symptoms
P=0.3688
Basophils
Days post onset of symptoms

Supplementary Figure S3. Correlation heatmaps of the max. level of immune parameters in individual patients **a**, and per group: **b,** viral clearance ≤ 21 days and **c,** viral clearance > 21 days.

a

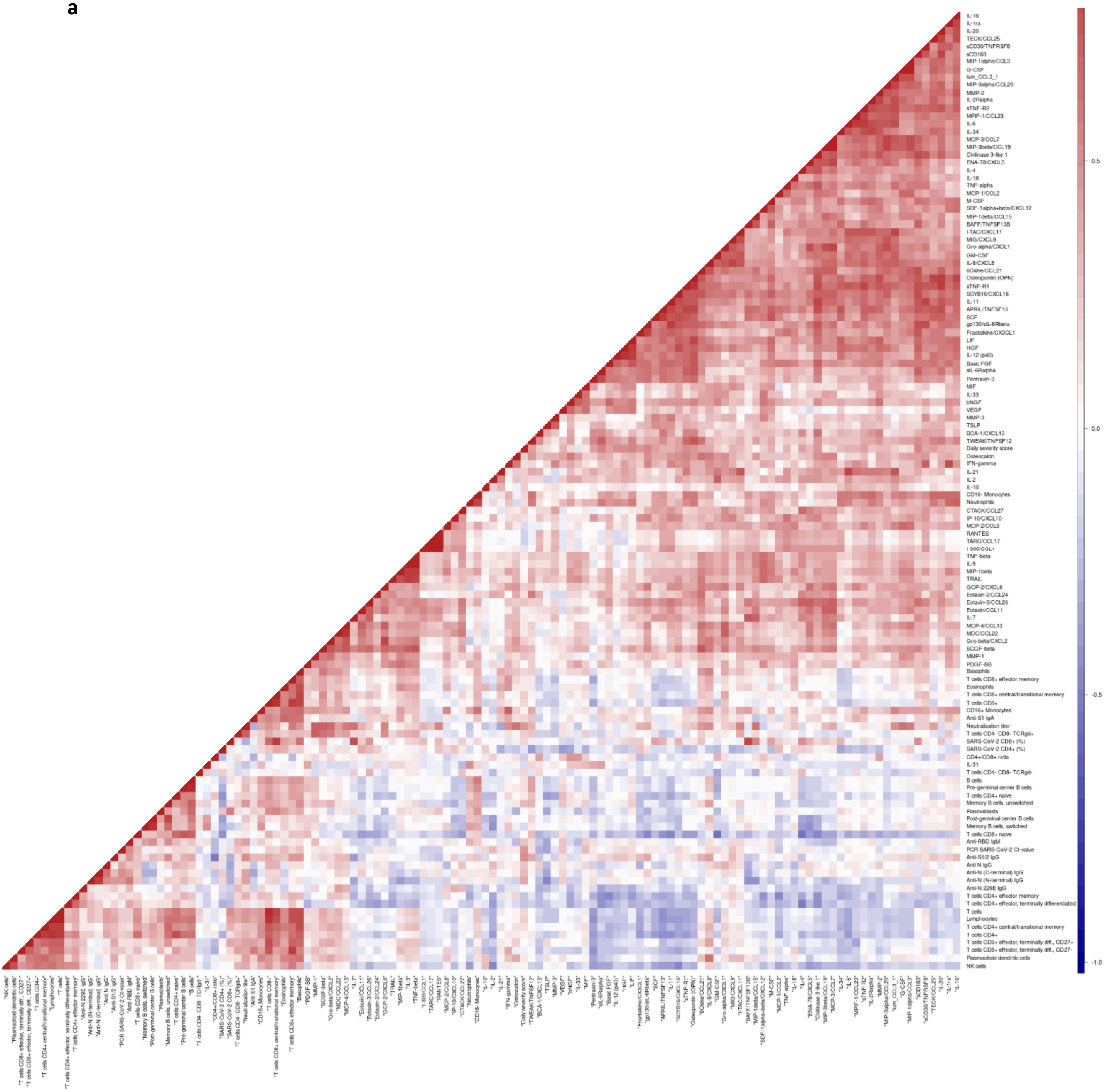

b

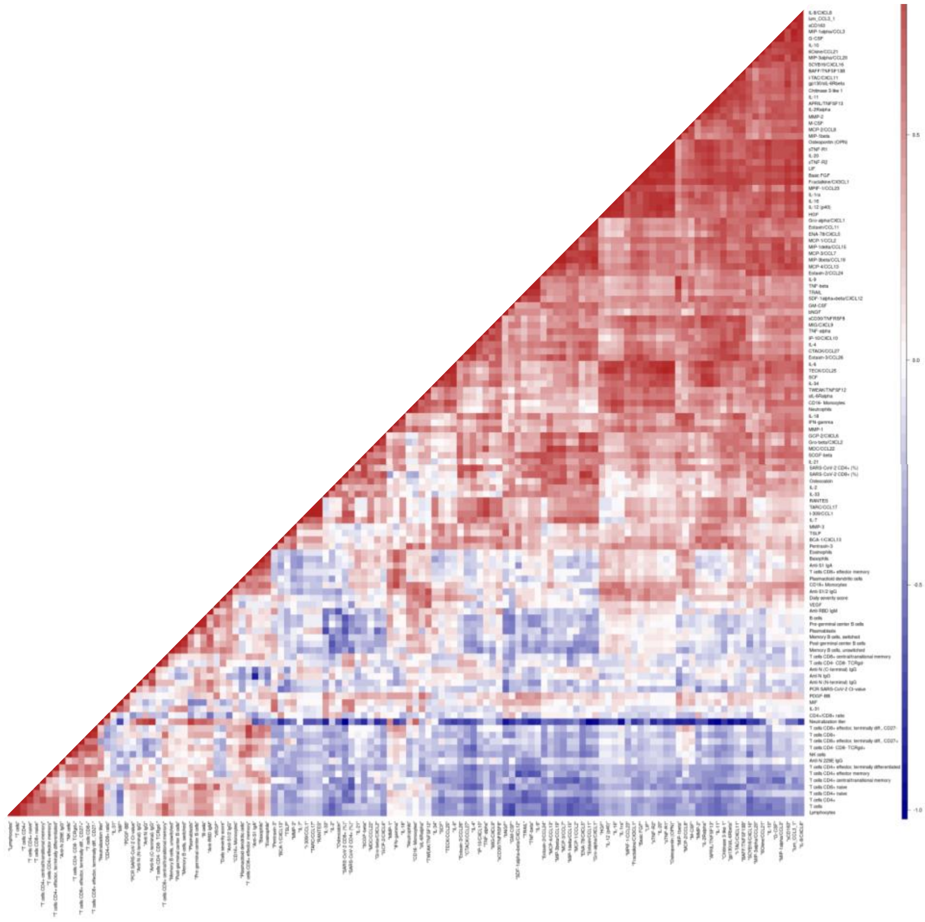

c

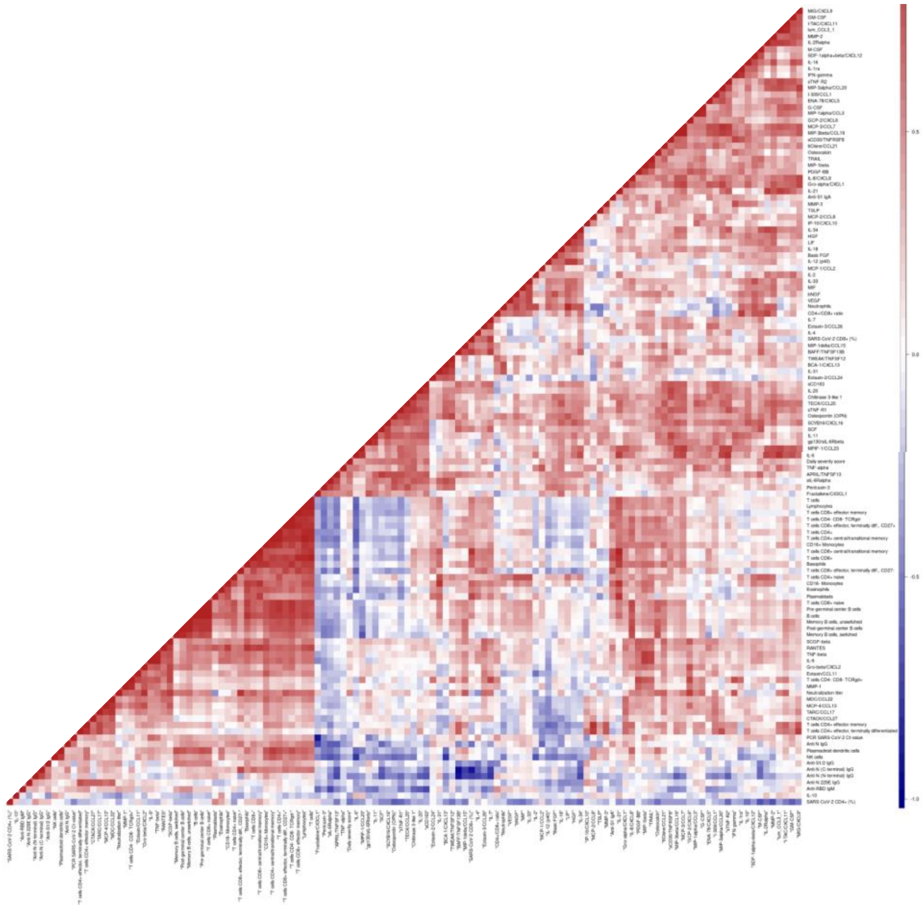

Supplementary Figure S4. Leukocyte subtypes identified by flow cytometry and their expression profiles reflecting the gating strategy used. A: APS (Automatic Population Separator) plots that use the information from all the parameters included in the file; B. Bi-parameter plots reflecting the expression profile for each of the markers used (Far-left: major leukocyte subsets, middle-left: major lymphocyte subsets, middle right: main T cell subsets; far-right: main B cell sunsets; DCs = dendritic cells, EM = effector memory cells, Eff = terminally differentiated effector cells, NK = natural killer, TM = transitional memory cells.

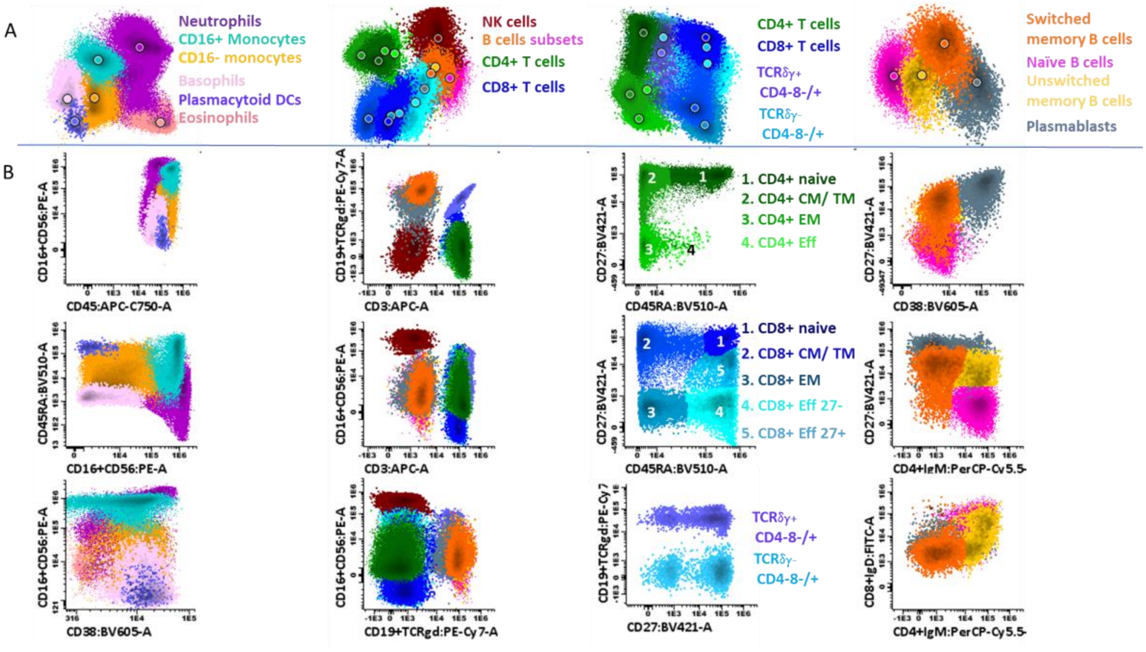

Supplement: Supplementary file 1 [file cells-11-02743-s001.zip › 04_Supplementary Figures S1-S4 2022-08-17_PDF.pdf]
